# A meta-analysis of berberine combined with brain-gut axis-targeted agents for diarrhoea-predominant irritable bowel syndrome

**DOI:** 10.3389/fphar.2026.1795170

**Published:** 2026-09-30

**Authors:** Nanxi Liu, Yahui Huang, Huixia Qiao, Yue Wang, Xiaoxu Liu, Jiayu Zhang

**Affiliations:** 1 Department of Spleen and Stomach Diseases, Xi’an Hospital of Traditional Chinese Medicine, Xi’an, China; 2 The First School of Clinical Medicine, Shaanxi University of Chinese Medicine, Xianyang, China

**Keywords:** berberine, brain-gut axis-targeted agents, IBS-D, meta-analysis, rct, systematic review

## Abstract

**Background:**

The prevalence of diarrhoea-predominant irritable bowel syndrome (IBS-D) is high and significantly impairs patient quality of life. Berberine, a naturally occurring isoquinoline alkaloid, shows promise in combination with brain-gut axis-targeted drugs for relieving abdominal pain, bloating, and diarrhoea in IBS-D. However, current clinical evidence remains insufficient to conclusively substantiate its efficacy.

**Aim of the study:**

Through a systematic literature review and meta-analysis, this work comprehensively evaluated the efficacy and safety of berberine in combination with brain-gut axis-targeted medications for the treatment of IBS-D, thereby providing evidence to inform clinical practice.

**Materials and methods:**

On 1 December 2025, a comprehensive search was conducted across ten databases, including PubMed, Web of Science (WOS), the Cochrane Library, and Embase, to identify randomized controlled trials (RCTs) evaluating berberine in combination with brain-gut axis-targeted medications. Two reviewers independently screened the literature according to predefined inclusion and exclusion criteria, followed by data extraction. The risk of bias tool developed by the Cochrane Collaboration was used to assess the methodological quality of the included studies, and data were analyzed using RStudio software. When two or more studies had comparable interventions and outcome measures, a meta-analysis was conducted, otherwise, a qualitative synthesis was performed. The certainty of evidence for each outcome was assessed using the Grading of Recommendations, Assessment, Development and Evaluation (GRADE) approach.

**Results:**

A total of 19 RCTs involving 1,489 participants were included in the analysis. Compared with the control groups, the intervention groups showed significant improvements in IBS-D symptoms and patient quality of life. Specifically, combination therapy incorporating berberine significantly increased the overall treatment response rate (odds ratio = 4.19, 95% confidence intervals (CI): 3.13–5.61). With respect to continuous outcomes, combination therapy significantly reduced overall symptom scores (standardized mean difference (SMD) = −3.47, 95% CI: −6.65 to −0.29), particularly reflected by reductions in abdominal pain and bloating severity, along with improvements in diarrhoea-related symptoms. Furthermore, no serious adverse events were reported across the included trials.

**Conclusion:**

Berberine combined with brain-gut axis-targeted medications shows promising efficacy and acceptable safety in the treatment of IBS-D. However, existing studies are limited by small sample sizes and methodological weaknesses, and the overall strength of evidence remains insufficient. Larger, rigorously designed RCTs are needed to further confirm its efficacy and safety.

**Systematic Review Registration:**

https://www.crd.york.ac.uk/PROSPERO/view/CRD420251238184, identifier CRD420251238184.

## Graphical Abstract

**Figure:**
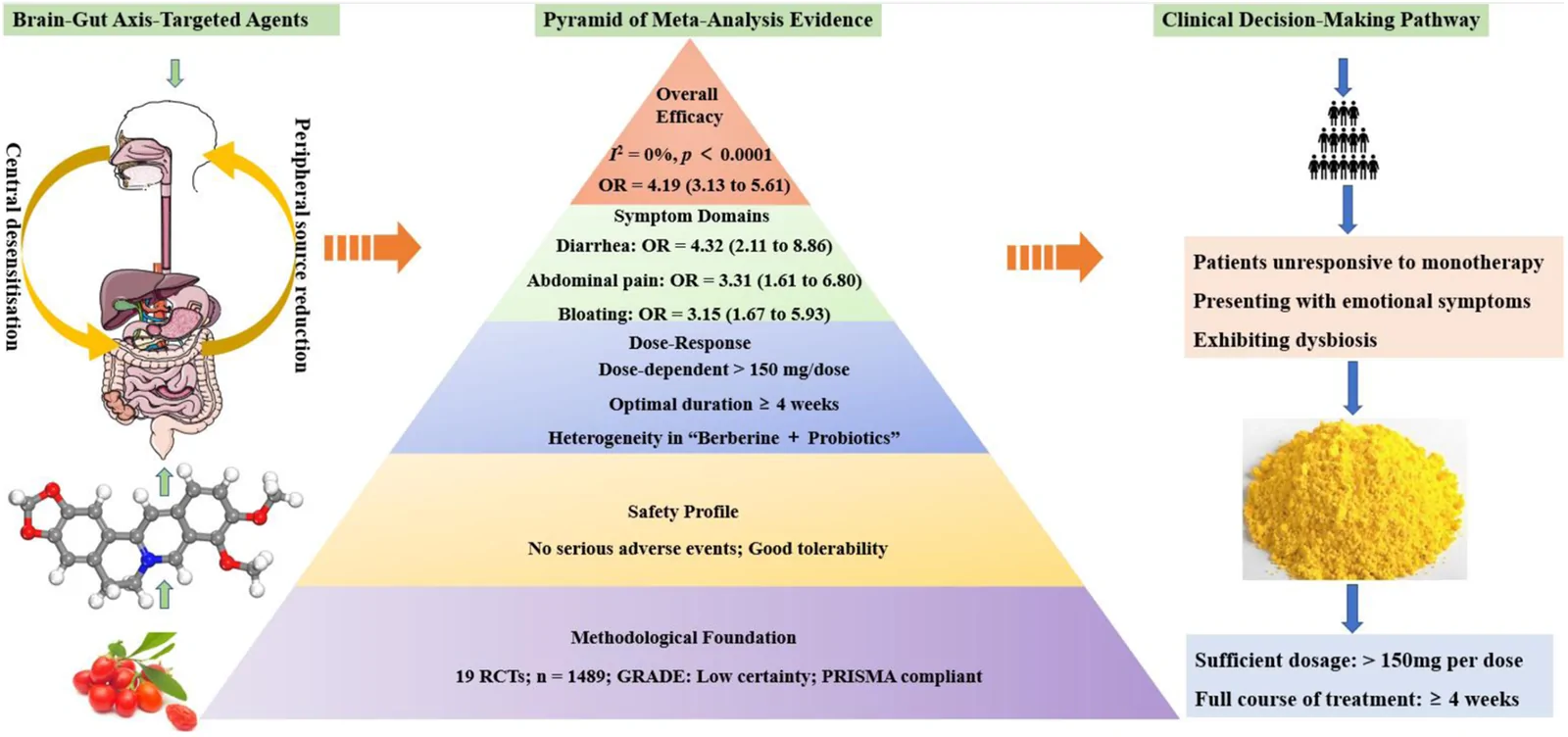

## Highlights

1. Combination therapy of berberine with brain-gut axis-targeted drugs demonstrated significant clinical efficacy in patients with IBS-D.
2. A dual-regulatory mechanism was proposed, involving synergistic effects of “peripheral-derived inhibition” and “central desensitization” to modulate brain-gut interactions at multiple levels.
3. Dose-response analysis established an effective threshold for berberine in the combination regimen (>150 mg per dose).
4. This work confirmed that combining berberine with brain-gut axis-targeted drugs did not increase the incidence of adverse events.

## 1 Introduction

Diarrhoea-predominant irritable bowel syndrome (IBS-D) is a functional gastrointestinal disorder primarily characterized by abdominal pain, diarrhoea, and altered bowel habits. It typically follows a chronic course with a high tendency for recurrence (Grayson, 2016; Greer and Sultan, 2025). Epidemiological studies indicate that IBS-D has a high prevalence (Mearin et al., 2016), which substantially impairs patient quality of life and increases healthcare burdens (Fadgyas-Stanculete et al., 2014; Faresjö et al., 2013; Black and Ford, 2020). However, the pathogenesis of IBS-D remains incompletely understood and is thought to involve multiple mechanisms, including altered intestinal motility, visceral hypersensitivity, gut-brain axis dysfunction, low-grade inflammation, and impaired intestinal barrier function (Ford et al., 2017; Tillisch et al., 2011). Consequently, single therapeutic approaches often fail to produce satisfactory clinical outcomes.

Against this backdrop, natural compounds with multi-target regulatory potential have emerged as a key focus in IBS-D research. Berberine is an isoquinoline alkaloid derived from traditional Chinese medicines such as *Coptis chinensis* Franch. (Huanglian) and *Phellodendron chinense* C.K. Schneid. (Huangbo) and exhibits multiple pharmacological effects, including anti-inflammatory activity, modulation of the gut microbiota, and improvement of intestinal barrier function (Wang et al., 2024). The broad-spectrum antimicrobial activity of berberine-containing plant extracts against multiple pathogens has been well documented (Salehi et al., 2019). In addition, study by Ehteshamfar et al. (2020) further highlighted the potential of berberine in regulating intestinal function and alleviating inflammatory responses. Several clinical studies (Sun et al., 2025; Wu et al., 2025; Liu et al., 2025; Habiba et al., 2025; Han et al., 2025; Yu et al., 2020) have investigated the use of berberine in the treatment of IBS-D or related diarrhoeal disorders and reported potential therapeutic benefits. However, the existing evidence is largely derived from studies with small sample sizes, and the findings remain inconsistent across studies.

In recent years, the brain-gut axis has been recognized as a key component in the pathogenesis of IBS-D (Lerner et al., 2017). This concept emphasizes bidirectional regulatory interactions among the central nervous system, enteric nervous system, gut immune system, and gut microbiota, which play a pivotal role in the development of visceral hypersensitivity, abnormal intestinal motility, and emotional disorders (Quigley, 2018). Consequently, pharmacological agents targeted brain-gut axis-related pathways have been increasingly incorporated into the clinical management of IBS-D and have demonstrated efficacy in alleviating symptoms such as diarrhoea and abdominal pain (Wei et al., 2024). However, monotherapy remains limited in its ability to comprehensively improve symptoms and achieve sustained long-term efficacy (Yuan et al., 2023).

Given the multifactorial pathogenesis of IBS-D, combination therapeutic strategies targeting multiple pathological pathways have gained increasing attention. The effects of berberine on intestinal function and inflammatory responses may complement those of brain-gut axis-targeted drugs, thereby enhancing overall therapeutic efficacy. Several recent studies (Chen et al., 2015; Di Pierro et al., 2020; Chen et al., 2022) have investigated the clinical efficacy of berberine or brain-gut axis-targeted pharmacotherapies for IBS-D. However, clinical evidence regarding their combined application remains limited, and existing findings lack systematic integration and quantitative evaluation.

This work employs systematic review and meta-analysis methodologies to comprehensively evaluate the clinical efficacy and safety of berberine combined with targeted brain-gut axis medications in the treatment of IBS-D. Relevant RCTs were systematically retrieved. Following rigorous literature screening and quality assessment, meta-analysis was conducted to quantitatively synthesize the effect sizes of combined treatment regimens on efficacy outcomes, including diarrhoea, abdominal pain, abdominal distension, and overall response rate. Concurrently, safety profiles were evaluated, including recurrence rates, and subgroup analyses were performed to investigate the effects of different drug combinations, dosages, and treatment durations on efficacy. Through systematic integration and quantitative analysis of existing clinical evidence, this work provides objective support for the clinical application of berberine combined with targeted brain-gut axis medications, aiming to inform clinical decision-making and personalized treatment strategies for IBS-D.

## 2 Research content and methods

### 2.1 Literature sources and retrieval methods

Guided by the PICOS (population, intervention, comparator, outcome, study design) framework, a comprehensive systematic search strategy was developed. Electronic searches were conducted across multiple databases, including the China National Knowledge Infrastructure (CNKI) full-text database, Wanfang Data, China Science and Technology Journal Database (VIPC), Chinese Biomedical Literature Database (CBM), PubMed, Web of Science (WOS), Cochrane Library, Embase, SinoMed, and Scopus. Supplementary searches using Google Scholar and backward citation tracking were performed to maximize retrieval of relevant studies. The search period covered from the inception of each database up to December 2025. Relevant clinical studies investigating the combined use of berberine and targeted brain-gut axis medications for treating IBS-D were collected. Search terms in both Chinese and English included “Irritable Bowel Syndrome”, “Diarrhoea-predominant”, “Berberine”, “Brain-gut axis”, “RCT”, “Traditional Chinese Medicine”, and “Chinese herbal medicine”. A “subject term + free-text term” strategy was applied, following the “P + I + S” retrieval framework.

### 2.2 Study registration

The research protocol has been registered with the International Prospective Register of Systematic Reviews (PROSPERO) under registration number CRD420251238184. The research report will adhere to the Preferred Reporting Items for Systematic Reviews and Meta-Analyses (PRISMA) guidelines (Moher et al., 2009), with research questions formulated based on the PICOS framework.

### 2.3 Inclusion and exclusion criteria

#### 2.3.1 Inclusion criteria

The inclusion criteria for RCTs were as follows: (a) The study design was an RCT that assessed berberine combined with targeted brain-gut axis medications for IBS-D treatment, with no restrictions on blinding. (b) The intervention group received combined therapy that included berberine, while the control group received probiotics, gastrointestinal regulators, berberine, or Jianpi compounds. (c) Research subjects met the diagnostic criteria for IBS-D according to Rome II (Drossman, 1999), Rome III (Drossman, 2006), or Rome IV (Sood and Ford, 2016), and were aged ≥18 years. (d) Studies employed a three-tier efficacy evaluation: “highly effective”, “effective”, and “ineffective”. (e) Primary outcomes included overall response rate, symptom scores, recurrence rate, adverse reactions, clinical symptom assessment post-intervention, abdominal pain score, diarrhoea score, bloating score, and response rates for abdominal pain, diarrhoea, and bloating. (f) Included studies utilized randomized allocation, provided complete data, and demonstrated balanced baseline characteristics.

#### 2.3.2 Exclusion criteria

The exclusion criteria were as follows: (a) NRCT or non-randomized prospective studies without a control group. (b) Studies with incomplete data, inappropriate statistical analyses, excessively high loss-to-follow-up rates, or missing key outcomes. (c) Studies with duplicate publications or redundant datasets. (d) Studies in which IBS-D was diagnosed without internationally recognized criteria (e.g., Rome II, III, or IV). (e) Clinical studies that did not exclude other organic intestinal or infectious diseases. (f) Studies in which the intervention protocol for the experimental group included primary therapeutic agents other than berberine. (g) Studies with outcome measures that were not aligned with the objectives of this meta-analysis. (h) Non-clinical literature, including animal studies, reviews, expert opinions, case reports, and theoretical discussions.

### 2.4 Quality assessment and data extraction

#### 2.4.1 Quality assessment

This work was conducted following the Cochrane Handbook for Systematic Reviews of Interventions (Version 6.1) (Higgins et al., 2011). Two reviewers independently assessed the methodological quality of the included RCTs using the Cochrane Risk of Bias tool. In the event of discrepancies between the evaluations provided by different reviewers, consensus was reached through consultation, with third-reviewer input sought where necessary.

#### 2.4.2 Data extraction

The assessment included random sequence generation, allocation concealment, blinding of participants and personnel, blinding of outcome assessment, incomplete outcome data, selective reporting, and other potential sources of bias. Additionally, the following study characteristics were extracted: first author, publication year, sample size, baseline patient characteristics (including age and disease duration), intervention details, treatment duration, outcome measures, and safety assessment data. For missing or unclear data, corresponding authors were contacted to request clarification or additional information. If no response was received, data were estimated when feasible or excluded from analysis.

### 2.5 Statistical processing

RStudio is an open-source software environment designed for statistical computing and graphics. Data processing and graphical representations were performed using R packages such as meta, ggplot2, dplyr, stringr, gt, and risk of bias within RStudio (version 4.5.1). During the meta-analysis, appropriate effect measures were chosen according to outcome types: odds ratio (OR) or risk ratio (RR) for dichotomous variables, and SMD for continuous variables. Heterogeneity was assessed using the chi-square test and the *I* ^2^ statistic, with 95% confidence intervals (CI) calculated at a significance level of α = 0.05. The degree of heterogeneity was quantified using the *I* ^2^ statistic. If *I* ^2^ ≤ 50% and *p* > 0.1, heterogeneity was considered low, and fixed-effects model was used. If *I* ^2^ > 50% or *p* ≤ 0.1, heterogeneity was considered high, and a random-effects model was applied to estimate effect sizes.

If substantial heterogeneity was detected and sufficient data were available, subgroup analyses, sensitivity analyses, or meta-regression analyses were performed. Subgroups were defined based on treatment regimens, combinations of berberine with other therapies, and berberine dosage. When heterogeneity could not be adequately explained, a descriptive analysis was performed. To assess the stability and reproducibility of the results, a sensitivity analysis was conducted by removing outliers to investigate the root cause. Pooled effect estimates were visualized using forest plots. Publication bias was assessed using funnel plot inspection, Egger’s linear regression test, and Begg’s rank correlation test. When funnel plot asymmetry or statistical tests indicated potential publication bias, the trim-and-fill method was applied to estimate and adjust for missing studies. A *p*-value <0.05 was considered statistically significant for all analyses.

## 3 Results and analysis

### 3.1 Search results and inclusion status

The flow diagram of study screening with reference to PRISMA 2020 statement was shown in Figure 1. A total of 2,061 articles were identified through systematic database searches, with an additional 82 articles identified through reference tracing. After removing duplicates, 1,250 articles remained for screening. Titles and abstracts were initially screened to exclude reviews, meta-analyses, case reports, irrelevant studies, non-RCTs, studies not meeting population criteria, and those with non-compliant interventions, after which full-text articles were assessed for eligibility. After full-text review, 63 articles were excluded due to incomplete data, absence of primary outcome measures, or failure to meet scale assessment criteria. Ultimately, 19 articles were included in the qualitative synthesis and meta-analysis (Yin et al., 2010; Fu et al., 2010; Zhao, 2013; Ou, 2005; Zhao and Ma, 2008; Zeng, 2004; Ji, 2006; Yu, 2012; Ji et al., 2020; Peng, 2010; Hua, 2008; Chen et al., 2008; Zhao and Zhu, 2013; He and Li, 2009; Chen, 2009; Zeng, 2012; Liang and Jiang, 2016; Wu, 2006; Zhu, 2006). In the intervention groups, berberine was combined with targeted brain-gut axis therapies: berberine combined with probiotics in eight studies, antidepressants in three studies, gastrointestinal regulators in four studies, and Jianpi compound decoctions in four studies. Control groups received probiotics, berberine, gastrointestinal regulators, or Jianpi compound decoctions, with treatment durations ranging from 2 to 12 weeks. A total of 1,489 participants were included, with 767 in the intervention groups and 722 in the control groups. The composition and major constituents of *Coptis chinensis* (Huanglian) are shown in Figure 2. The baseline characteristics of the included RCTs are summarized in Table 1.

**FIGURE 1:**
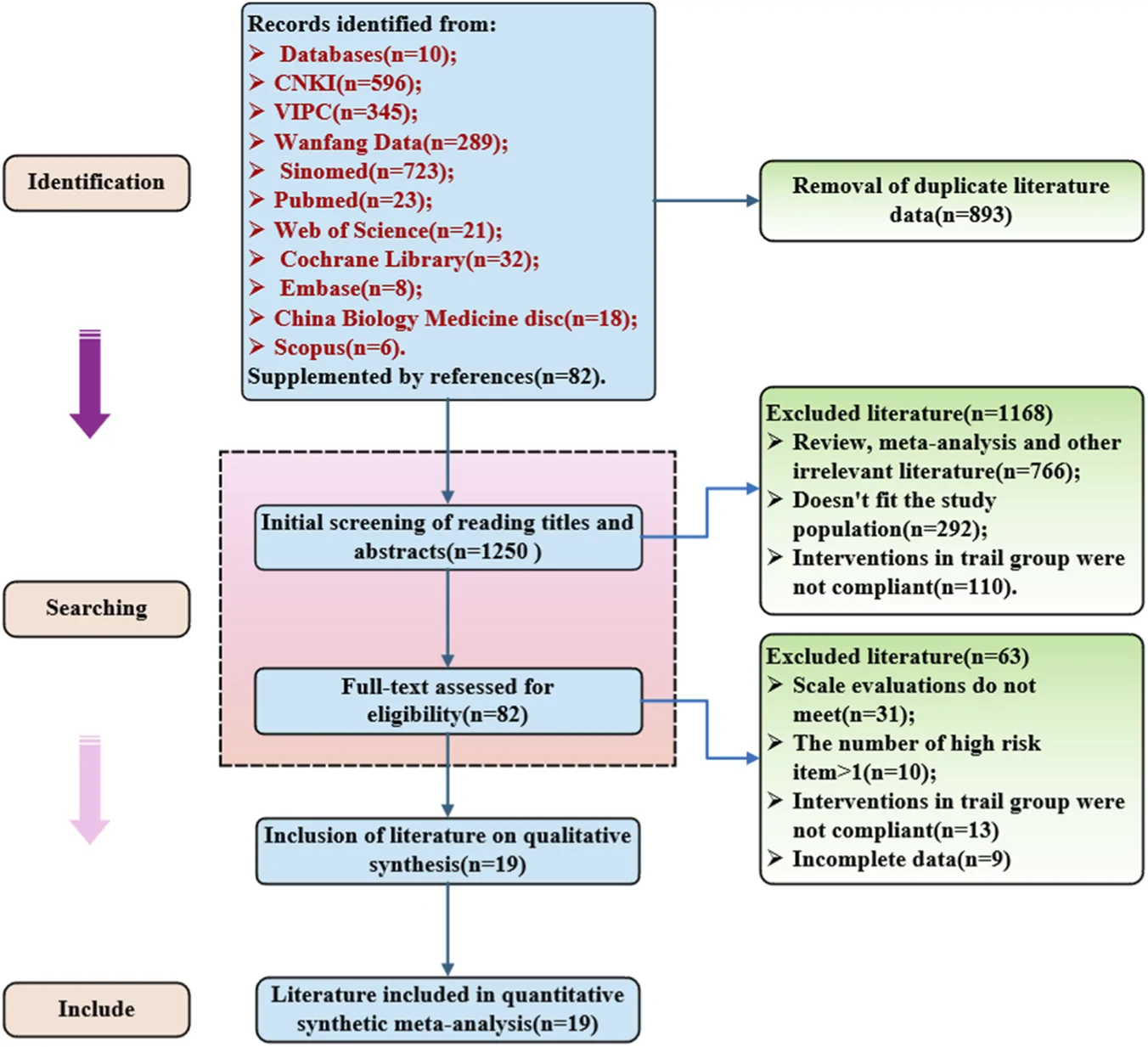
Flow diagram of work screening.

**FIGURE 2:**
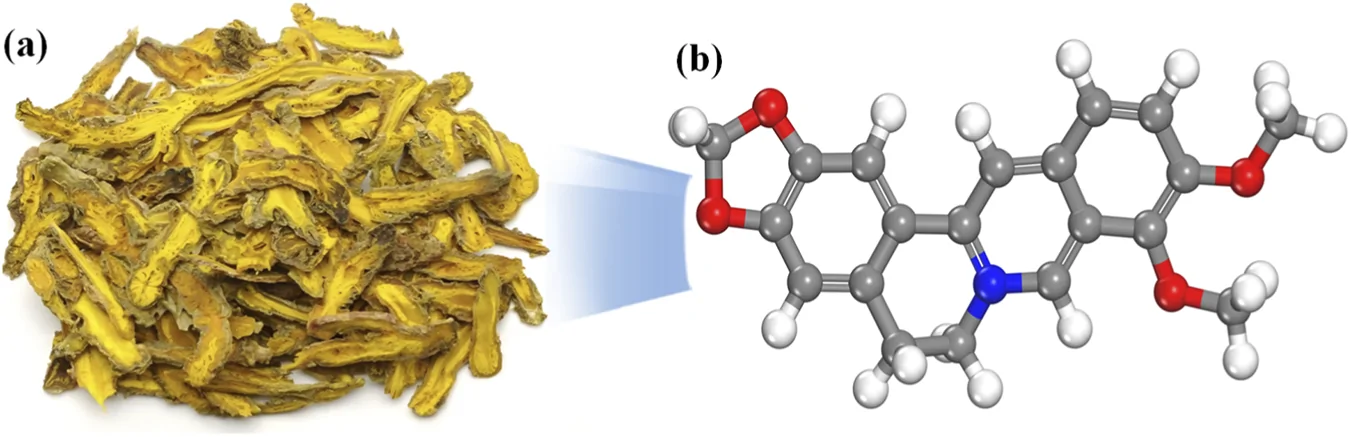
*Coptis chinensis* (Huanglian) and its principal constituents comprise: **(a)** *Coptis chinensis* (Huanglian) (the primary botanical source of berberine); **(b)** The principal composition of berberine (C_20_H_18_NO_4_^+^).

**TABLE 1:** Characteristics of included trials.

Author (year)	N	Male/Female	Age (T/C)	Course of IBS-D (T/C)	Therapy (T)	Therapy (C)	Treatment duration	Outcomes	Safe or not	Random or not
Yin et al. (2010)	T:40 C:40	18/22 16/24	40.5/43.5	0.5–8.5/0.5–8 (years)	Berberine combined with live combined *bacillus subtilis* and *enterococcus* faecium enteric-coated capsules	Berberine	3 weeks	①	Yes	Yes
Fu et al. (2010)	T:60 C:60	25/35 33/27	43.5/42.3	NR	Berberine combined with flupentixol and melitracen tablets	Berberine	12 weeks	①④	Yes	Yes
Zhao (2013)	T:40 C:40	NR	18–68	NR	Bupi Yiqi decoction combined with berberine	Berberine	8 weeks	①②③④	Yes	Yes
Ou RX (2005)	T:45 C:45	NR	38.6	0.5–12 (years)	Paroxetine combined with berberine	Berberine	4 weeks	①	Yes	Yes
Zhao and Ma, 2008	T:38 C:26	15/23 9/11	38.2 ± 9.8/41.3 ± 10.1	4.1 ± 1.3/5.1 ± 1.2 (years)	Berberine combined with bifidobacterium quadruple viable tablets	Bifidobacterium quadruple viable tablets	4 weeks	①④	Yes	Yes
Zeng (2004)	T:50 C:35	13/17 14/14	45.2 ± 13.7/42.2 ± 18.8	5.95 ± 0.81/6.12 ± 0.87 (years)	Berberine combined with *lactobacillus* complex	*Lactobacillus* complex	4 weeks	①②④⑤	Yes	Yes
Ji (2006)	T:48 C:48	NR	42 ± 7.8	NR	Berberine combined with montmorillonite powder	Bifidobiogen-Livzon combined with oryzanol	3 weeks	①④	Yes	Yes
Yu (2012)	T:36 C:36	20/16 23/13	41.6 ± 5.7/43.2 ± 6.2	3–16/2–15 (years)	Berberine combined with compound glutamin entresoluble capsules	Compound glutamin entresoluble capsules	2 weeks	①②④⑨⑩⑪	Yes	Yes
Ji et al. (2020)	T:38 C:38	16/22 18/20	43.1 ± 3.8/42.3 ± 4.1	14.9 ± 4.0/15.2 ± 3.1 (months)	Compound berberine combined with bifidobacterium quadruple viable tablets	Compound berberine	8 weeks	①②④	Yes	Yes
Peng (2010)	T:32 C:31	19/13 17/14	38.15 ± 11.63/37.94 ± 10.57	5.59 ± 5.31/37.94/±10.57 (years)	Jiawei Tongxie Yaofang combined with berberine	Jiawei Tongxie Yaofang	4 weeks	①②④⑥⑦⑧	Yes	Yes
Hua (2008)	T:68 C:68	21/47 19/49	51/52.5	3.5/3 (years)	Compound berberine combined with bifico, pinaverium bromide, and doxepine	Bifico, pinaverium bromide, and doxepine	2 weeks	①②③④	Yes	Yes
Chen et al. (2008)	T:30 C:30	NR	36.2	1–8 (years)	Berberine combined with bifico	Bifico	2 weeks	①	Yes	Yes
Zhao and Zhu (2013)	T:43 C:43	18/25 20/23	32.6/33.8	2.4/2.8 (years)	Berberine combined with pinaverium bromide	Pinaverium bromide	4 weeks	①	Yes	Yes
He and Li. (2009)	T:28 C:28	12/16 13/15	41.4 ± 7.5/40.7 ± 6.3	3.4 ± 1.5/3.6 ± 1.1 (years)	Berberine combined with trimebutine maleate	Trimebutine maleate	3 weeks	①④	Yes	Yes
Chen (2009)	T:32 C:32	20/44	34.5	0.5–6 (years)	Berberine combined with bifidobacterium quadruple viable tablets	Bifidobacterium quadruple viable tablets	2 weeks	①	Yes	Yes
Zeng (2012)	T:39 C:39	17/22 18/21	40.4 ± 5.8/39.6 ± 5.4	4.6 ± 1.2 4.1 ± 1.5	Berberine combined with Tongxie Yaofang with modifications	Trimebutine maleate capsule	4 weeks	①	Yes	Yes
Liang and Jiang (2016)	T:32 C:32	14/18 15/17	40.2/39.5	More than 1 year	Berberine combined with compound eosinophil-lactobacillus tablets	Compound eosinophil-lactobacillus tablets	4 weeks	①④	Yes	Yes
Wu (2006)	T:30 C:30	13/17 16/14	34.33 ± 13.61/33.33 ± 12.88	NR	Modified shenling baizhu powder combined with berberine	Berberine	4 weeks	①②⑥⑦⑧	Yes	Yes
Zhu (2006)	T:38 C:21	15/23 8/13	42.19/43.56	2.53/2.39 (years)	Berberine, bifidobiogen-livzon combined with flupentixol and melitracen tablets	General drugs	8 weeks	①⑨⑩⑪	Yes	Yes

NR, Not Report.

Indicators of outcomes (① Overall efficacy rate; ② Symptom score; ③ Recurrence rate; ④ Adverse reactions; ⑤ Assessment of clinical symptoms following intervention; ⑥Abdominal pain score; ⑦ Diarrhoea score; ⑧ Bloating score; ⑨ Abdominal pain efficacy rate; ⑩ Diarrhoea efficacy rate; **⑪** Bloating efficacy rate).

### 3.2 Assessment of methodological quality

A total of 19 RCTs were included. All trials reported baseline patient characteristics and provided detailed descriptions of intervention protocols and treatment regimens. However, the methodological quality of the included trials varied according to the risk of bias assessment. In the domain of random sequence generation, approximately eighty-five percent the RCTs (n = 16) were rated as low risk of bias. One trial was rated as high risk, while two trials mentioned randomization without specifying the method and were thus judged to have an unclear risk of bias. Thirty percent of the RCTs (n = 6) were assessed as low risk of bias for allocation concealment. Regarding blinding of participants and personnel, seventy percent of the trials (n = 13) were assessed as high risk due to the nature of the treatment regimens. For blinding of outcome assessment, fifteen percent of the trials (n = 3) were assessed as low risk of bias. Twenty percent of the trials (n = 4) selectively reported outcome measures and were thus judged to have an unclear risk of bias. Forty percent of the included RCTs (n = 8) showed no risk of bias in other domains. The results of the risk of bias assessment are presented in Figures 3, 4.

**FIGURE 3:**
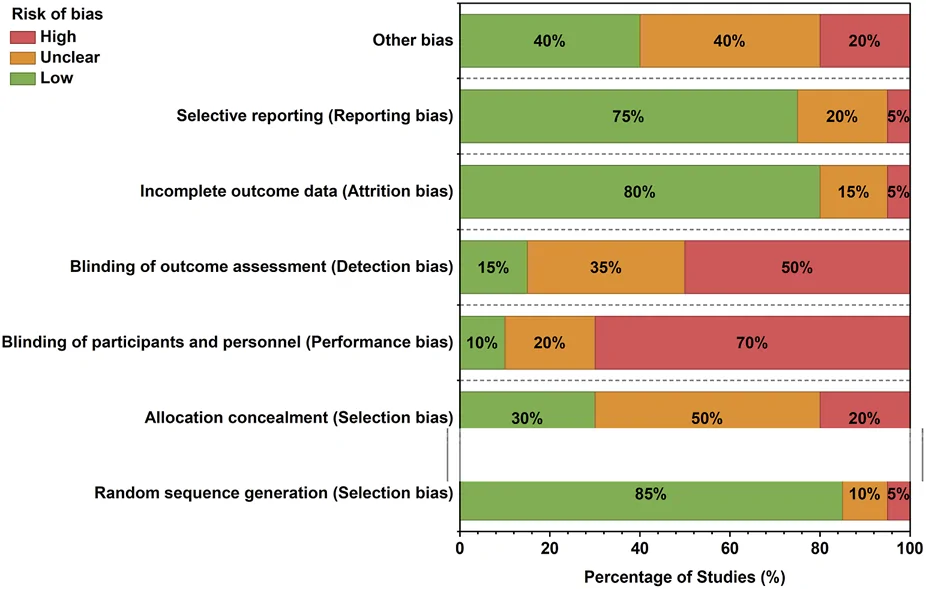
Risk of bias graph.

**FIGURE 4:**
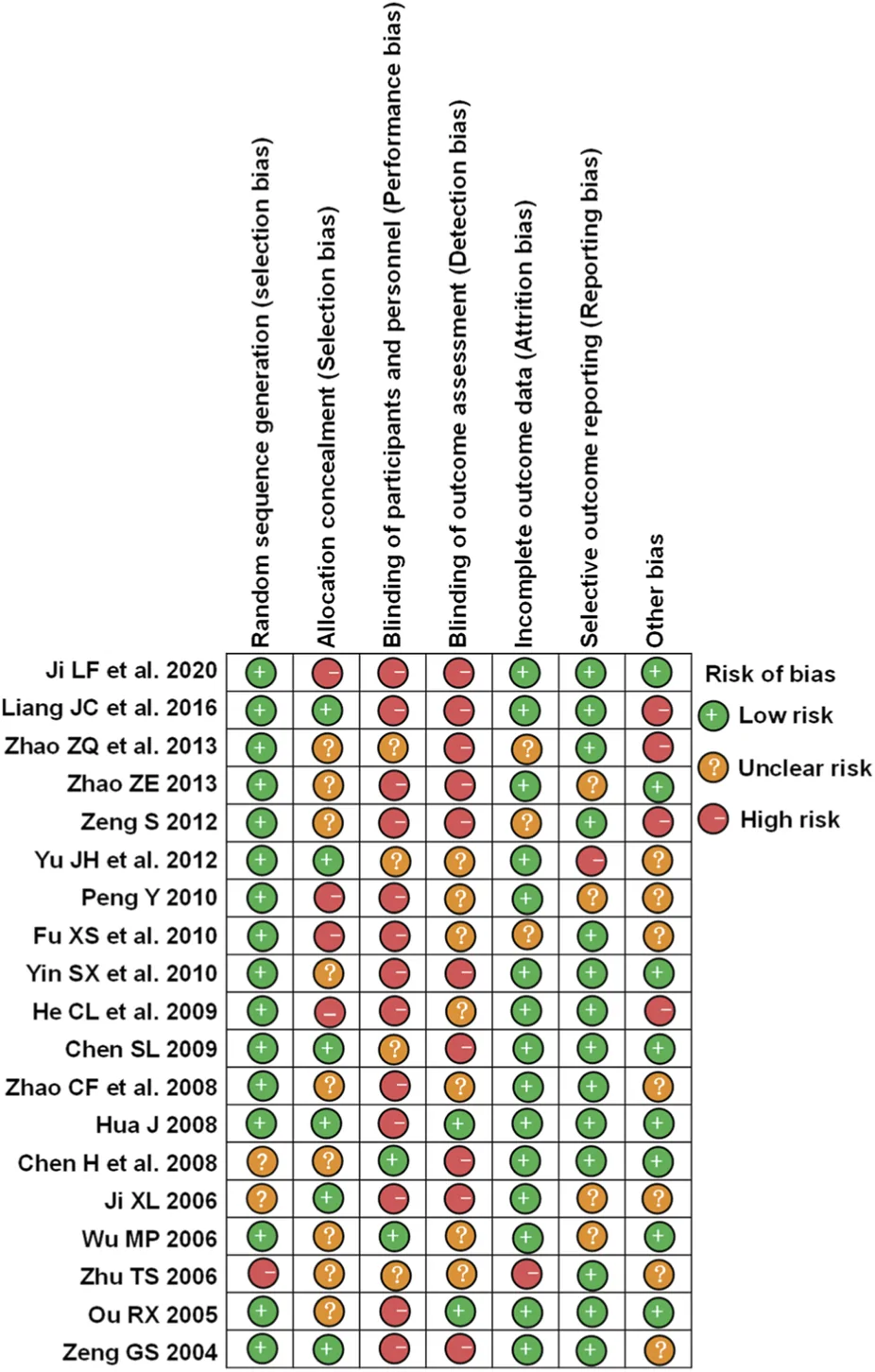
Risk of bias summary.

### 3.3 Statistical analysis

#### 3.3.1 Overall efficiency

All 19 included RCTs evaluated the efficacy of berberine in combination with targeted brain-gut axis medications for the treatment of IBS-D. Each trial reported the overall response rate as an outcome measure, with 767 participants in the combination-therapy groups and 722 in the monotherapy groups. The meta-analysis showed no heterogeneity across studies (*I* ^2^ = 0.0%, *p* = 0.9416), indicating a high level of consistency among the included trials. Therefore, a fixed-effects model was applied for the pooled analysis. The combination therapy demonstrated significantly greater efficacy than monotherapy (OR = 4.19, 95% CI: 3.13–5.61), with a highly significant overall effect (z = 9.65, *p* < 0.0001) (Figure 5), indicating that combination therapy for IBS-D substantially improves the overall response rate.

**FIGURE 5:**
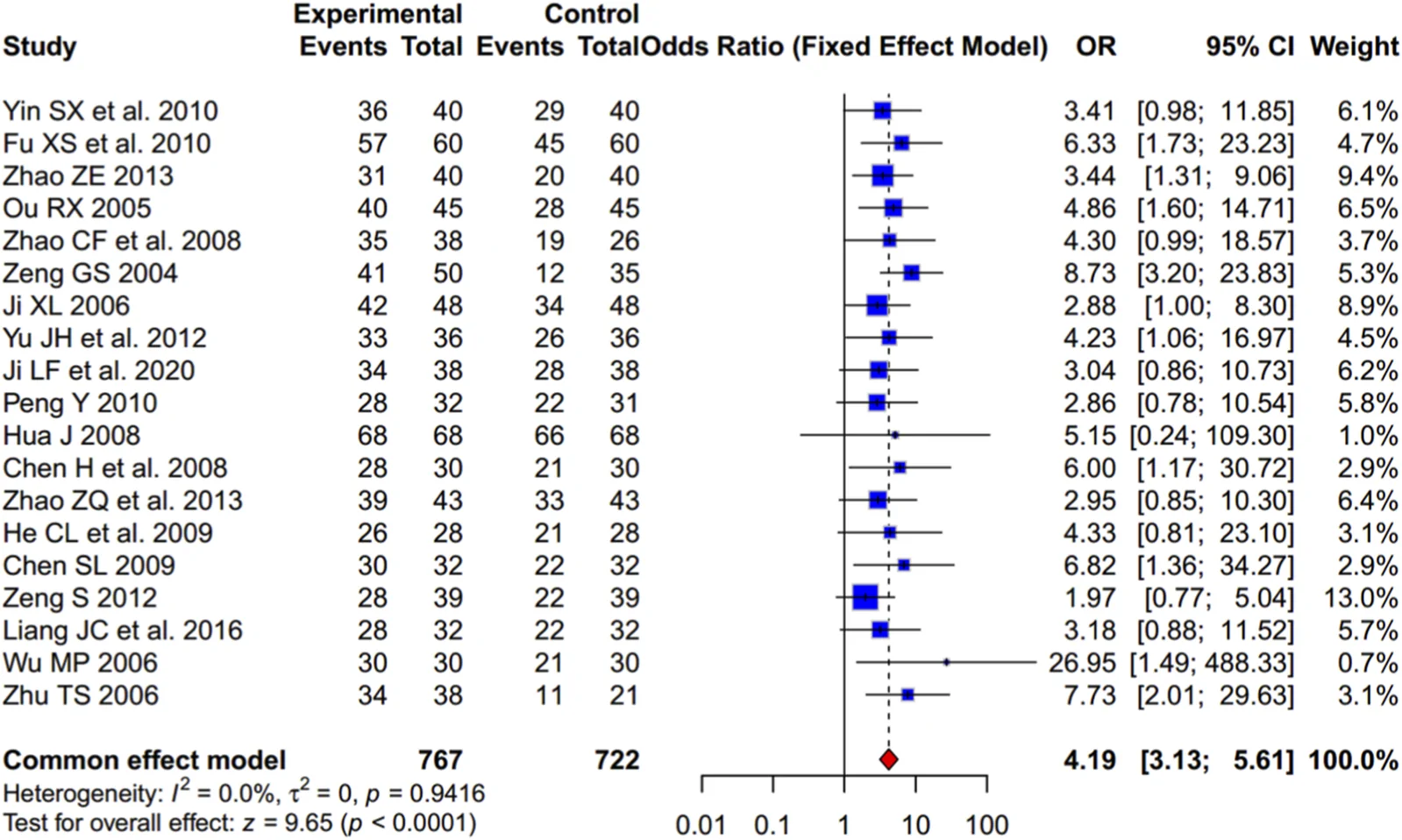
Forest plot comparing the overall response rate of berberine combined with brain-gut axis-targeted drugs versus monotherapy.

#### 3.3.2 Overall symptom scores

Two RCTs (Ji et al., 2020; Wu, 2006) systematically evaluated the efficacy of berberine combined with brain-gut axis-targeted agents in improving overall symptom scores. The treatment groups included 68 patients who received berberine plus brain-gut axis-targeted medication, while the monotherapy groups also included 68 patients. Substantial heterogeneity was observed between studies (*I* ^2^ = 96.4%, τ^2^ = 5.0674, *p* < 0.0001), which may be related to differences in symptom scoring criteria, baseline characteristics, subjective assessment, and inter-rater variability. Therefore, a random-effects model was applied for the meta-analysis, using the SMD as the effect size. Both RCTs showed significantly lower symptom scores in the combination-therapy groups than in the monotherapy groups (n = 136, SMD = −3.47, 95% CI: −6.65 to −0.29) (Figure 6).

**FIGURE 6:**
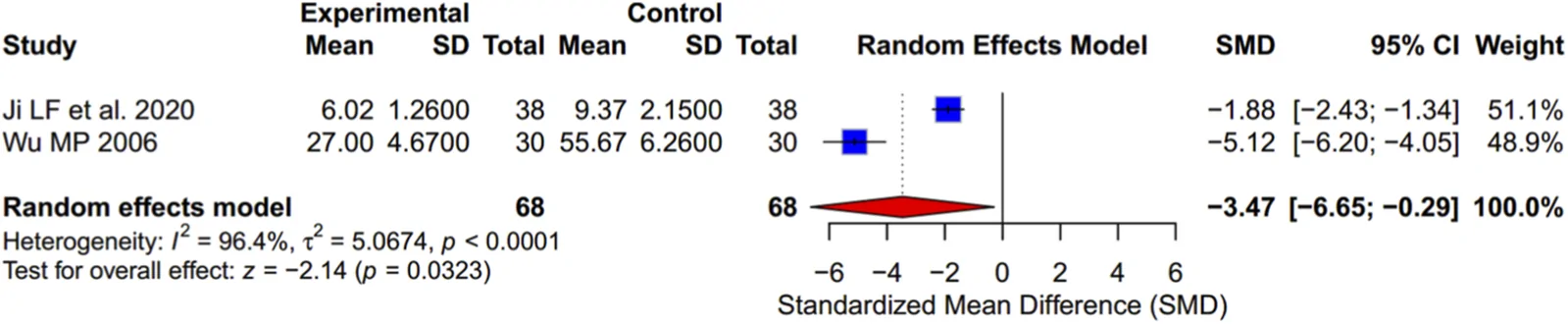
Forest plot comparing berberine combined with brain-gut axis-targeted therapy versus monotherapy in reducing overall symptom scores.

#### 3.3.3 Effective rate for diarrhoea

A total of four RCTs were included (Yu, 2012; Zhu, 2006; Peng, 2010; Wu, 2006) that evaluated the efficacy of berberine combined with targeted brain-gut axis therapy in improving diarrhoea symptoms. The combination-therapy groups included 136 patients, whereas the monotherapy groups included 118 patients. The heterogeneity test showed low between-study heterogeneity (*I* ^2^ = 17.0%, τ^2^ = 0.1355, χ3 2 = 3.61, *p* = 0.3065), and a fixed-effects model was therefore used for the meta-analysis. Compared with monotherapy, combination therapy resulted in significantly greater improvement in diarrhoea symptoms (n = 254, OR = 4.32, 95% CI: 2.11 to 8.86, z = 3.9976, *p* < 0.001) (Figure 7).

**FIGURE 7:**
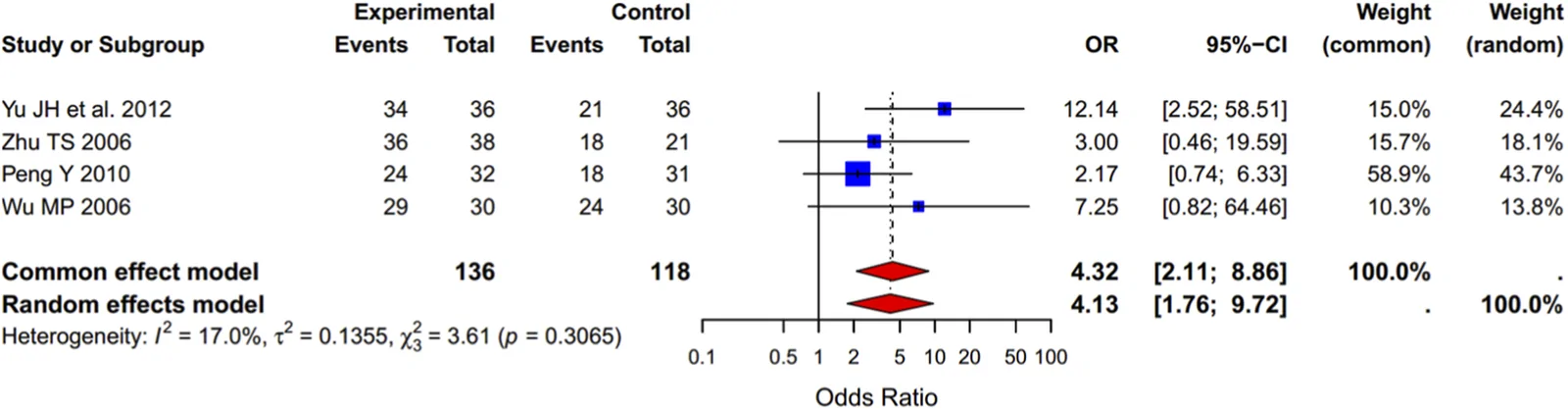
Forest plot comparing the efficacy of berberine combined with brain-gut axis-targeted therapy versus monotherapy in improving diarrhoea symptoms.

#### 3.3.4 Efficacy rate for abdominal pain

A total of four RCTs were included (Yu, 2012; Zhu, 2006; Peng, 2010; Wu, 2006) that evaluated the efficacy of berberine combined with brain-gut axis-targeted therapy in alleviating abdominal pain. The combination-therapy groups comprised 136 patients, whereas the monotherapy groups included 118 patients. As heterogeneity across studies was low (*I* ^2^ = 0.0%, τ^2^ = 0, χ2 3 = 2.41, *p* = 0.4921), a fixed-effects model was applied for the pooled analysis. The results showed that combination therapy provided a significant benefit in reducing abdominal pain (n = 254, OR = 3.31, 95% CI: 1.61 to 6.80, z = 3.2568, *p* = 0.00113) (Figure 8).

**FIGURE 8:**
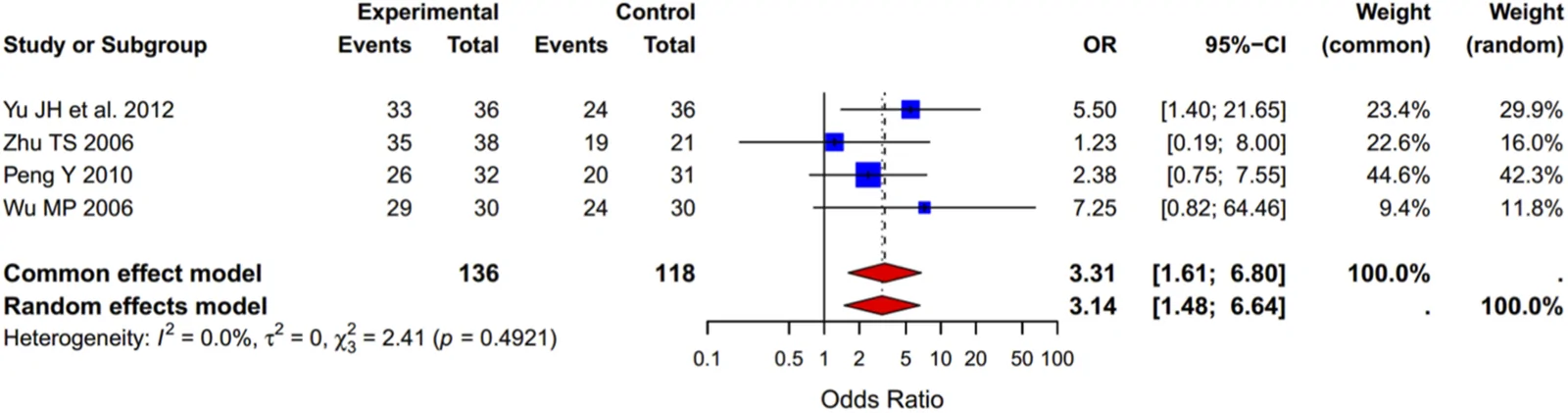
Forest plot comparing the efficacy of berberine combined with brain-gut axis-targeted therapy versus monotherapy in alleviating abdominal pain.

#### 3.3.5 Efficacy rate for abdominal distension

A total of four RCTs were included (Yu, 2012; Zhu, 2006; Peng, 2010; Wu, 2006) that evaluated the efficacy of berberine combined with brain-gut axis-targeted therapy in alleviating abdominal distension. The combination-therapy groups comprised 136 patients, whereas the monotherapy groups comprised 118 patients. Low between-study heterogeneity was observed (*I* ^2^ = 30.5%, χ3 2 = 4.32, *p* = 0.2292), supporting the use of a fixed-effects model for pooling. Combination therapy produced significantly greater improvement in abdominal distension than monotherapy (n = 254, OR = 3.15, 95% CI: 1.67–5.93) (Figure 9).

**FIGURE 9:**
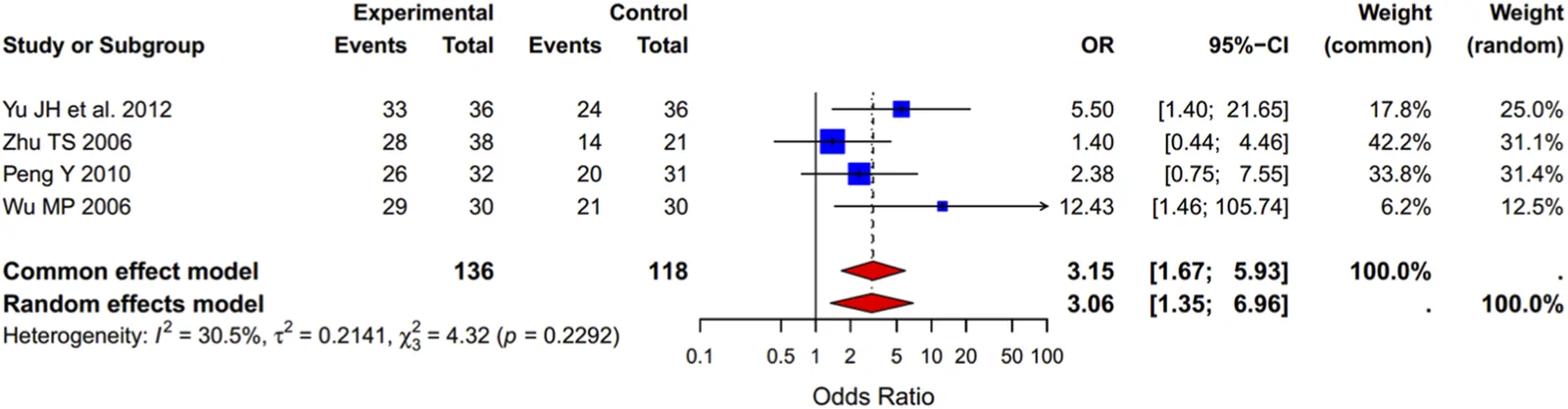
Forest plot comparing the efficacy of berberine combined with brain-gut axis-targeted therapy versus monotherapy in alleviating abdominal distension.

#### 3.3.6 Recurrence rate

Two RCTs (Zhao, 2013; Hua, 2008) were included, comparing disease recurrence rates between a combination therapy group (berberine plus brain-gut axis-targeted drug) and a monotherapy group. Both groups were followed for 2 months, each comprising 108 patients. The recurrence rate in the combination therapy group was 7.44% (8/108), significantly lower than 22.22% (24/108) in the monotherapy group. No heterogeneity was detected between studies (*I* ^2^ = 0.0%, χ2 1 = 0.07, *p* = 0.7971), allowing use of a fixed-effects model for meta-analysis. Results showed that combination therapy significantly reduced recurrence risk compared to monotherapy (n = 216, OR = 0.28,95% CI: 0.12 to 0.66, *p* = 0.0034) (Figure 10).

**FIGURE 10:**
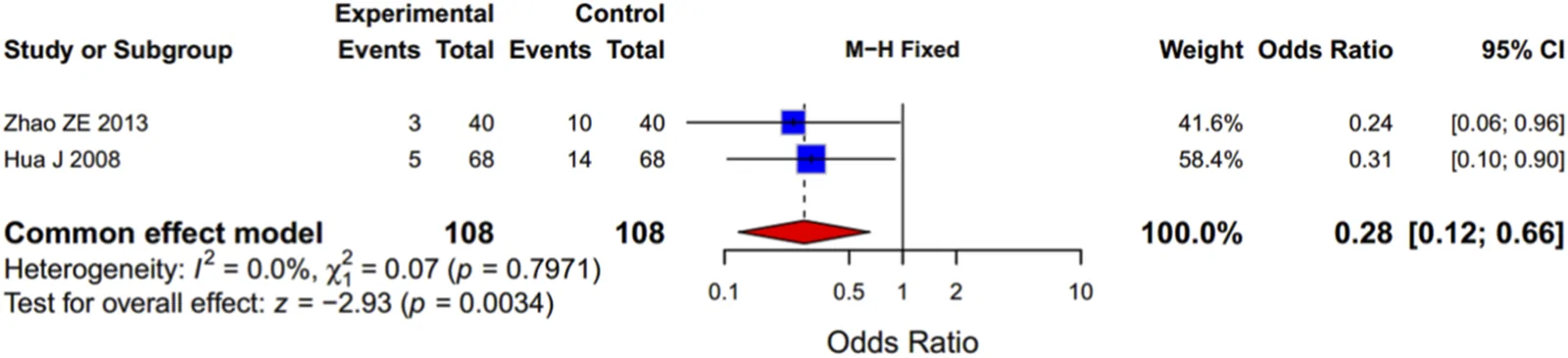
Forest plot comparing berberine combined with brain-gut axis-targeted therapy versus monotherapy in reducing recurrence rates.

#### 3.3.7 Adverse reactions

Eleven RCTs (Fu et al., 2010; Zhao, 2013; Zhao and Ma, 2008; Zeng, 2004; Ji, 2006; Yu, 2012; Ji et al., 2020; Peng, 2010; Hua, 2008; He and Li, 2009; Liang and Jiang, 2016) assessed adverse reactions in a total of 912 patients. Ten RCTs reported no adverse reactions. One RCT (Hua, 2008) reported adverse events: in both the berberine plus brain-gut axis-targeted drug group and the monotherapy group, two patients experienced dry mouth and three reported mild nausea. In addition, mild dizziness occurred in ten patients in the combination-therapy group and nine in the monotherapy group.

#### 3.3.8 Publication bias assessment

Publication bias for the overall clinical response rate was assessed using a funnel plot, with the logarithm of the OR (log OR) on the x-axis and the standard error (SE) on the y-axis. Quantitative analyses included the trim-and-fill method, Egger’s linear regression test, and Begg’s rank correlation test. The funnel plot (Figure 11) showed a symmetrical distribution, with the 19 included trials evenly distributed on both sides of the pooled effect size (OR = 4.19), indicating no evidence of publication bias. No heterogeneity was detected among studies (*I* ^2^ = 0.0%, τ^2^ = 0, *p* = 0.94), and the pooled effect size was statistically significant (z = 9.65, *p* < 0.0001). These results suggest minimal susceptibility to publication bias, thereby supporting the reliability of the conclusions.

**FIGURE 11:**
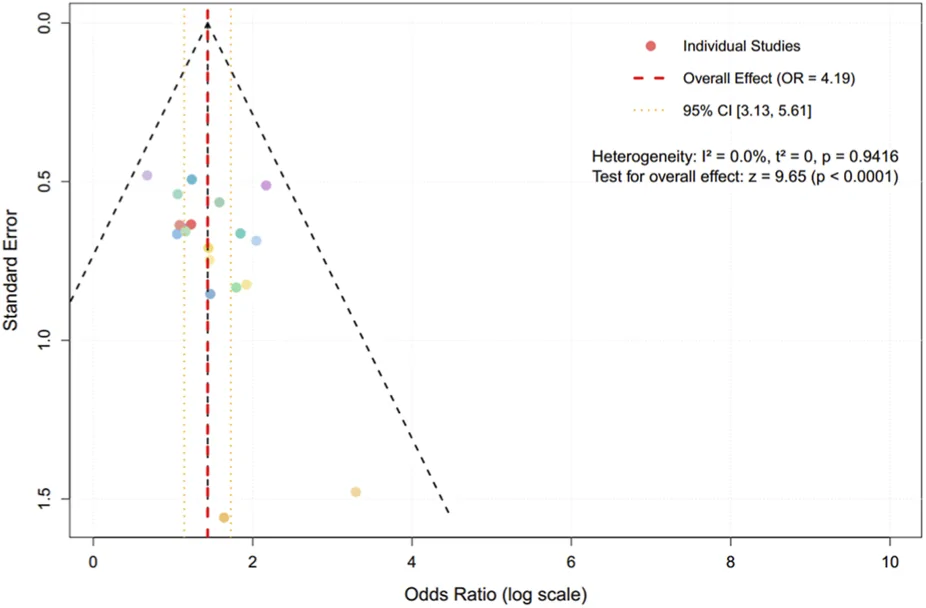
Funnel plot for overall response rates in clinical treatment.

Funnel plot analysis showed that most included studies clustered within the dark grey area, indicating high statistical significance (*p* < 0.01), whereas notable asymmetry was observed in the lighter grey areas corresponding to *p* < 0.05 and *p* < 0.10, suggesting potential publication bias. The trim-and-fill analysis identified six potentially missing studies in the lighter grey area. After imputation, the pooled effect size decreased from OR = 4.19 (95% CI: 3.13–5.61) to OR = 3.24 (95% CI: 2.49–4.21) (Figure 12). Although the effect size decreased, it remained highly significant, with no heterogeneity detected (*I* ^2^ = 0%), indicating consistent results across studies. This confirms that despite the absence of small-sample negative studies, the overall direction and clinical significance of the effect remain unchanged.

**FIGURE 12:**
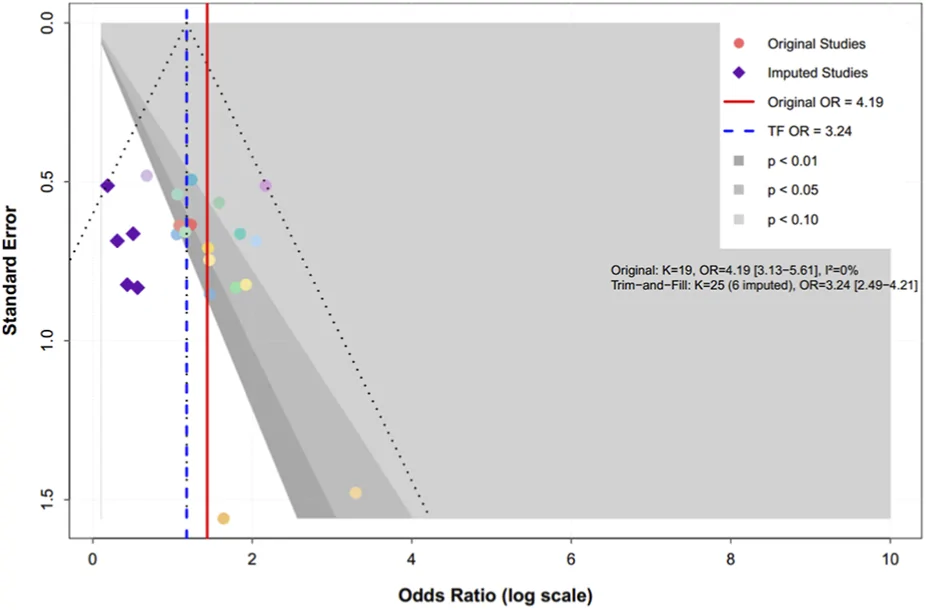
Funnel plot of overall clinical response rate for the trim-and-fill method.

Egger’s linear regression test yielded an intercept of 0.62 (*p* = 0.15), as illustrated in Figure 13a, indicating no statistically significant publication bias. However, Begg’s rank correlation test yielded a Kendall’s tau of 0.357 (*p* = 0.03), as illustrated in Figure 13b, suggesting the possible presence of minor publication bias. The discrepancy between these tests may be attributed to the uneven distribution of small-sample studies. Nevertheless, visual inspection of the funnel plot suggests that publication bias is unlikely to substantially affect the overall conclusions.

**FIGURE 13:**
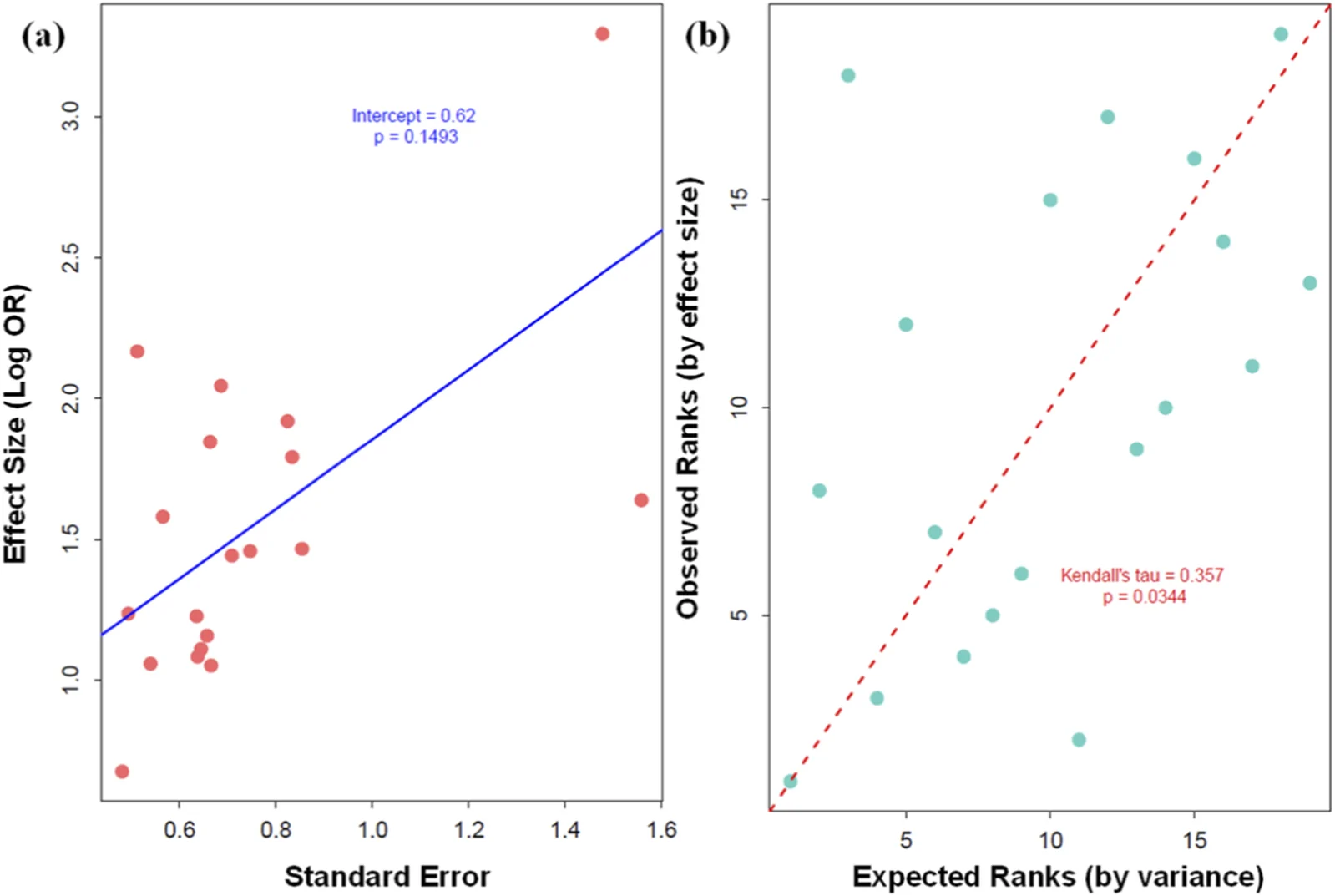
Assessment of publication bias for overall clinical response rates: **(a)** Egger’s linear regression plot; **(b)** Begg’s rank correlation plot.

Sensitivity analyses of the overall clinical response rate were conducted to assess the robustness and reproducibility of the findings. Across different analyses, pooled ORs remained consistent: 4.19 (95% CI: 3.13–5.61) in the original analysis; 3.77 (95% CI: 2.73–5.20) after excluding outliers; and 3.82 (95% CI: 2.80–5.22) when including only large-sample RCTs. The random-effects model yielded an effect size of 4.08 (95% CI: 3.03–5.47). Restricting the analysis to high-quality RCTs reduced the effect size to 2.59 (95% CI: 1.32–5.06), which remained statistically significant (Figure 14a). Leave-one-out sensitivity analyses demonstrated that exclusion of any single study caused minimal changes to the pooled effect size, with variations ranging from −6.1% to 7.9% (Figure 14b). Heterogeneity was consistently absent (*I* ^2^ = 0.0%), indicating that no individual study or analytical approach disproportionately influenced the findings, thereby confirming the reliability of the results.

**FIGURE 14:**
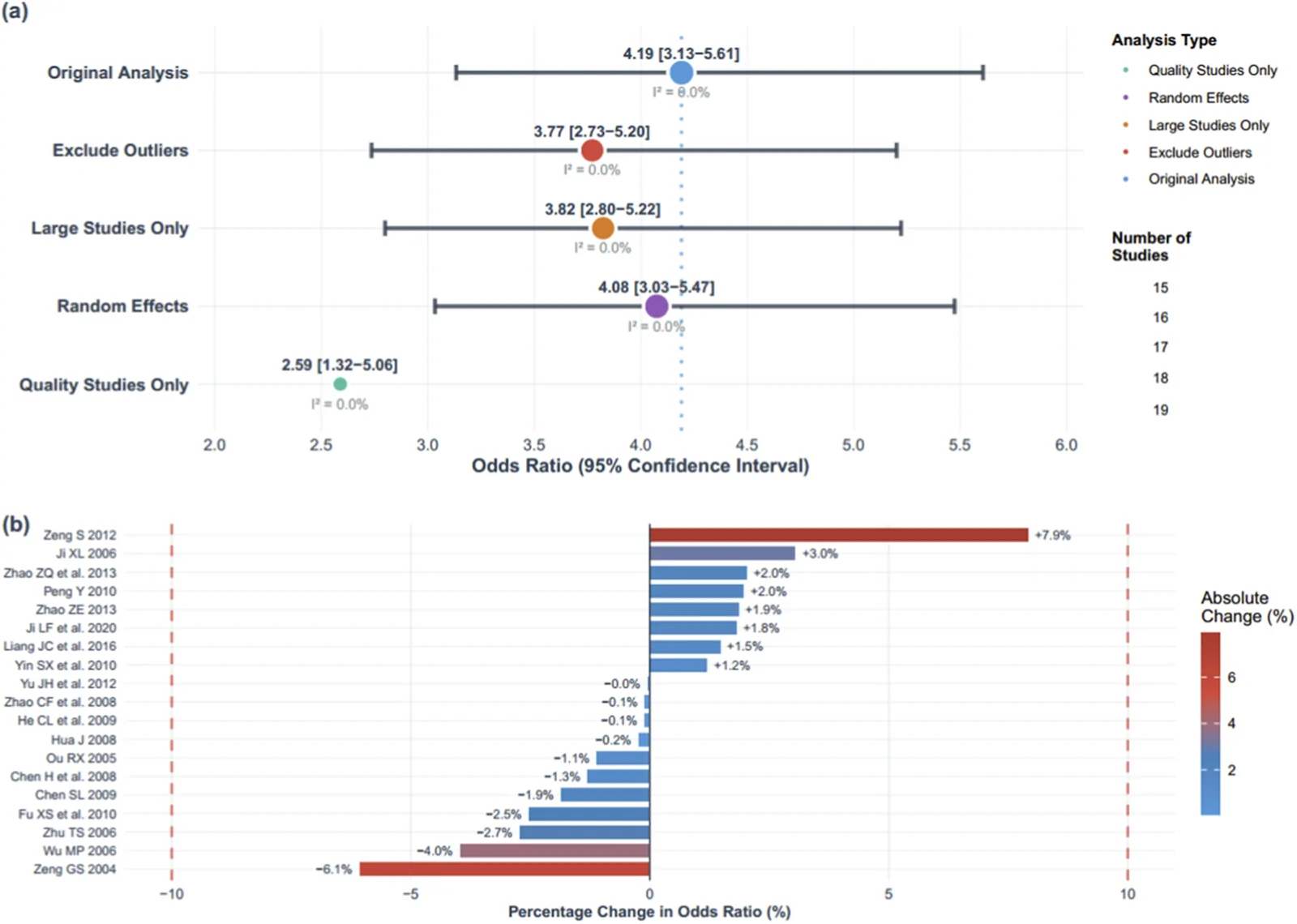
Stability testing of the overall clinical response rate: **(a)** Sensitivity analysis; **(b)** leave-one-out analysis.

Due to the limited number of trials (n = 4) assessing abdominal pain, diarrhea, and bloating, funnel plot analysis for publication bias could not be performed, potentially limiting the robustness of the conclusions. Both the Begg’s test (*p* = 0.17–0.86) and Egger’s test (*p* = 0.15–1.00) yielded non-significant results (Table 2), suggesting that the positive findings are unlikely to be influenced by publication bias, thereby supporting the robustness of the conclusions.

**TABLE 2:** Begg and Egger publication bias tests.

Meta-analysis	Studies	Begg *p* value	Egger *p* value	Bias risk
Abdominal pain	4	0.174	0.146	low risk
Diarrhea	4	0.861	1.000	low risk
Abdominal distension	4	0.400	0.497	low risk

#### 3.3.9 Regression analysis

Meta-regression analysis showed no significant trend in treatment efficacy over the 16-year period (β = −0.0087, 95% CI: 0.97 to 1.01, *p* = 0.37) (Figure 15). Although the RR decreased by approximately 0.87% annually, this trend was not statistically significant. Publication year accounted for approximately 49% of the heterogeneity among studies (*I* ^2^ = 49%), indicating that the remaining variation is attributable to other factors.

**FIGURE 15:**
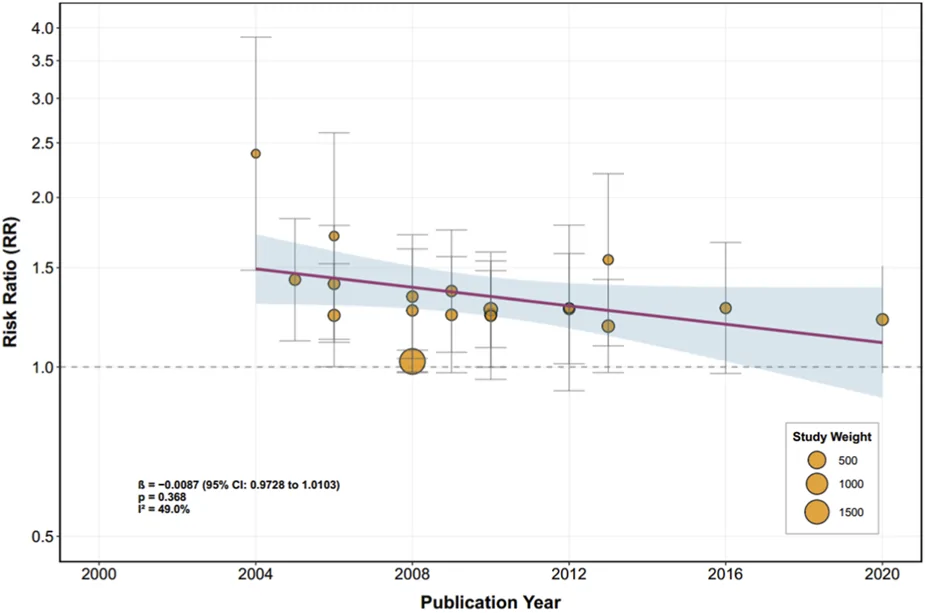
Meta-regression analysis of publication year and treatment efficacy. Note: The size of each point represents its weight (inverse variance), and the solid line indicates the regression line.

#### 3.3.10 Subgroup analysis

Subgroup analyses by treatment regimen revealed that the overall random-effects model demonstrated significant efficacy for combination therapy (RR = 1.31, 95% CI: 1.22–1.42), despite substantial heterogeneity (*I* ^2^ = 86.6%) (Figure 16). The heterogeneity was mainly driven by the two-week treatment subgroup (*I* ^2^ = 93.1%), whereas the three-week (*I* ^2^ = 0%), four-week (*I* ^2^ = 25.9%), and eight-week (*I* ^2^ = 34.8%) subgroups exhibited lower within-group heterogeneity. This suggests that treatment duration is a significant contributor to the overall heterogeneity. Although differences in efficacy across treatment duration subgroups were not statistically significant (*p* = 0.54), point estimates indicated a plateau in efficacy as treatment duration increased from 2 weeks (RR = 1.23) to 12 weeks (RR = 1.27). The 4-week treatment subgroup included the largest number of RCTs and produced the most consistent results (RR = 1.33). In summary, berberine combination therapy demonstrated efficacy across various treatment durations, with higher heterogeneity observed in short term (2 weeks) treatment and lower heterogeneity in medium to long term (3–12 weeks) treatment.

**FIGURE 16:**
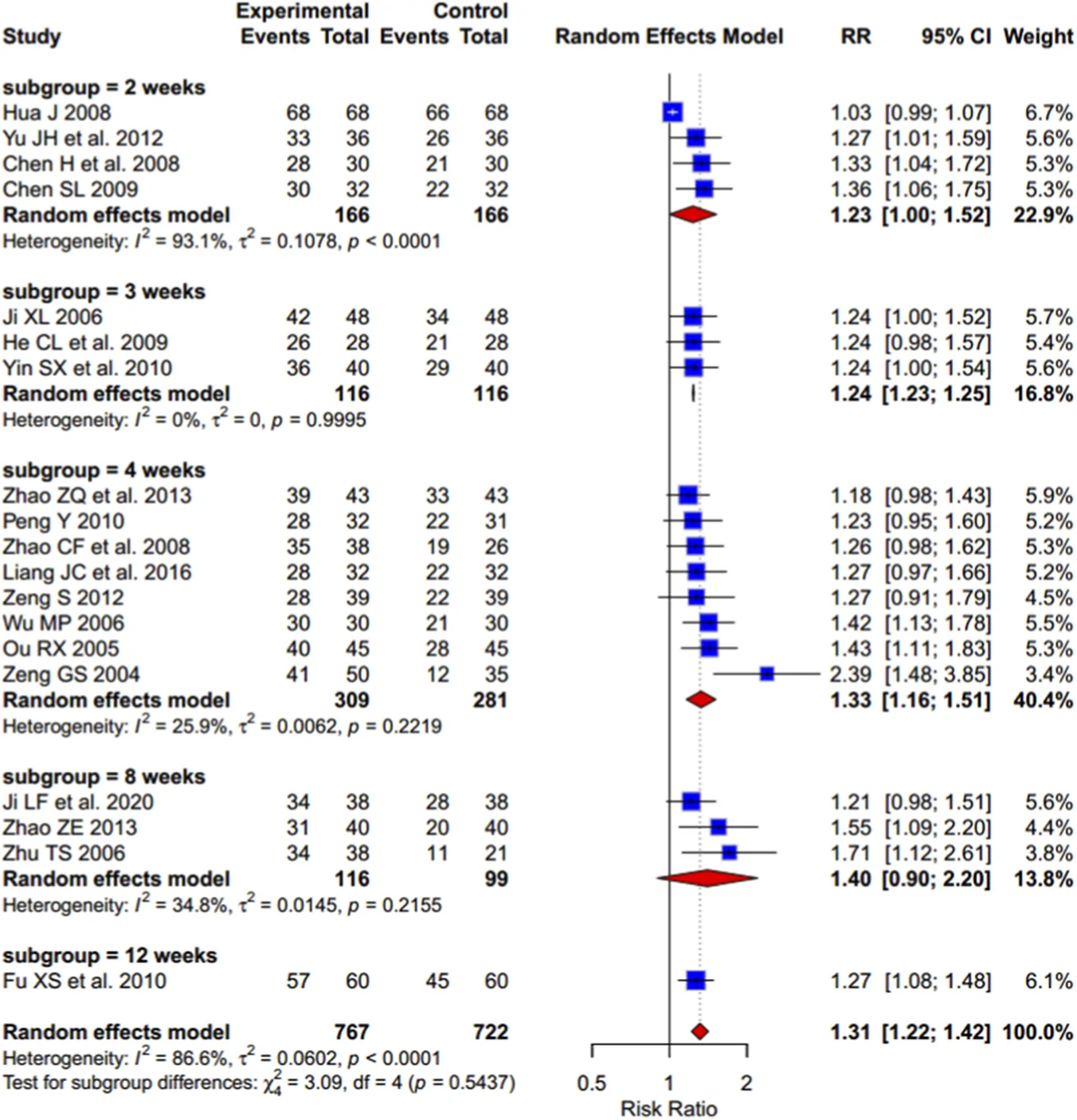
Subgroup analysis forest plot comparing differences in overall response rates between treatment regimens.

Subgroup analyses based on different berberine combination regimens showed that pooled results from the random-effects meta-analysis significantly improved the clinical response rate (RR = 1.31, 95% CI: 1.22–1.42), with substantial heterogeneity among studies (*I* ^2^ = 86.6%, *p* < 0.0001) (Figure 17). Further analysis indicated that heterogeneity mainly originated from the “berberine + probiotics” subgroup (*I* ^2^ = 93.6%), whereas the other three subgroups (“berberine + antidepressants”, “berberine + gastrointestinal regulators”, and “berberine + Jianpi compound decoction”) showed low heterogeneity (*I* ^2^ = 0–13.4%). Although efficacy differences between subgroups were not statistically significant (*p* = 0.11), the effect estimate for the “berberine + probiotics” subgroup was higher (RR = 1.32), and its individual studies showed considerable variation in effect sizes. This pattern suggests that this subgroup is a potential primary driver of the high overall heterogeneity. These results support the overall efficacy of combination therapy; however, interpretation of findings from the “berberine + probiotics” subgroup is limited by its high heterogeneity.

**FIGURE 17:**
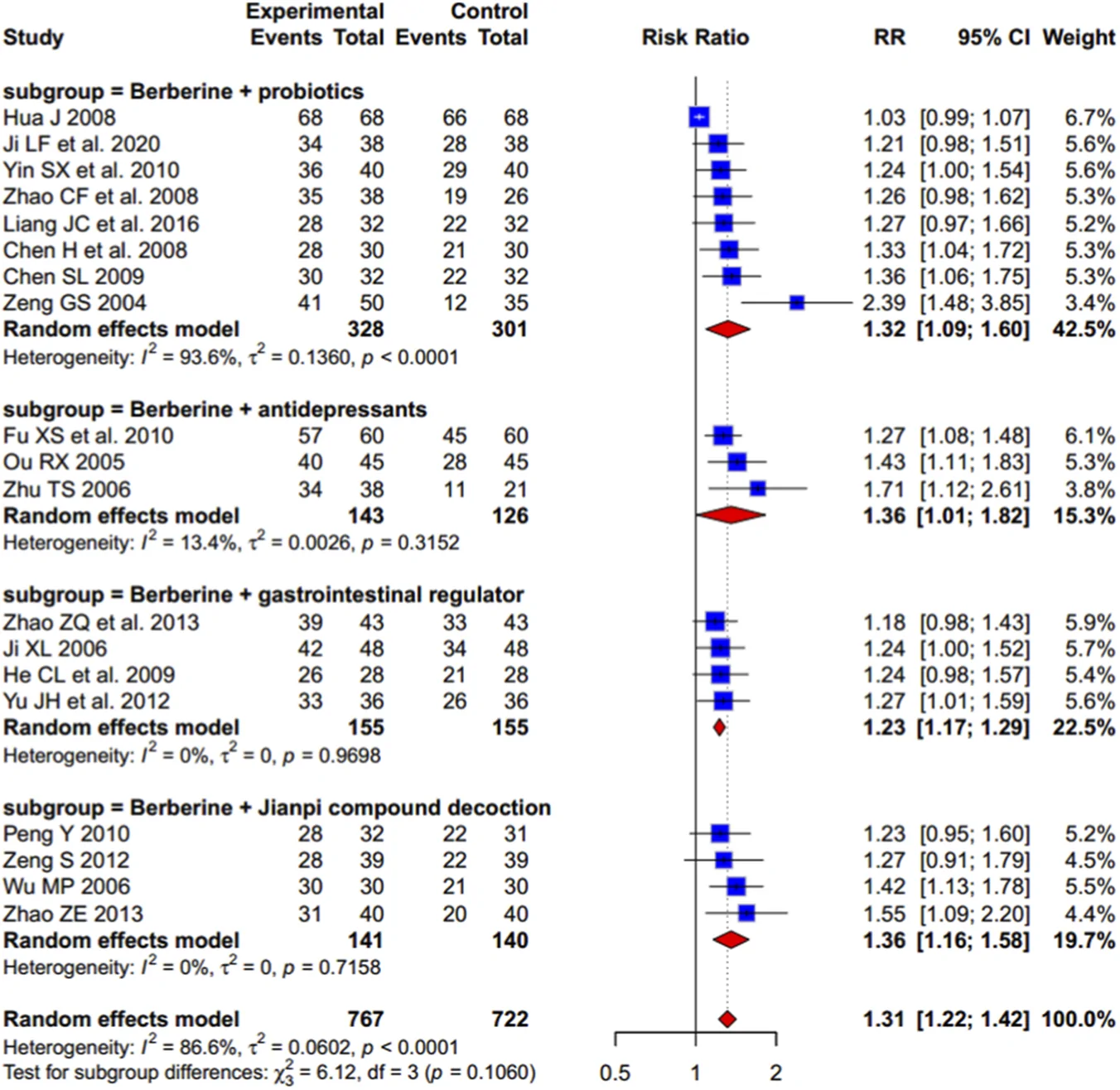
Forest plot for subgroup analysis comparing differences in overall response rates among various combination regimens of berberine.

Subgroup analysis stratified by single-dose berberine demonstrated a threshold-dependent dose-response relationship in IBS-D. The low-dose group (51 ∼ 150 mg per dose) comprised two studies and exhibited moderate heterogeneity (*I* ^2^ = 52.2%, *p* = 0.1480). The pooled effect estimate was RR = 1.04 (95% CI: 0.99 to 1.08) (Figure 18), suggesting that this dose may be below the minimum effective threshold for a clinically meaningful effect. At doses < 151 mg, the medium-dose group (151 ∼ 250 mg) comprised nine studies and exhibited negligible heterogeneity (*I* ^2^ = 0%, *p* = 0.9707). The pooled effect estimate was RR = 1.31 (95% CI: 1.20 to 1.42), indicating a statistically significant and consistent effect. The high-dose group (251 ∼ 350 mg) comprised eight studies and demonstrated efficacy (RR = 1.23, 95% CI: 1.13 to 1.33), but exhibited substantial heterogeneity (*I* ^2^ = 73.5%, *p* = 0.0004), indicating increased between-study variability. Overall, the pooled analysis demonstrated substantial heterogeneity (*I* ^2^ = 70.7%, *p* < 0.0001), with a summary RR of 1.10 (95% CI: 1.07 to 1.14). Differences between subgroups were statistically significant ( $\chi_{2}^{2}$ = 30.75, df = 2, *p* < 0.0001). These findings suggest that the dose-response relationship may plateau beyond the minimum effective dose. However, increased variability at higher doses indicates that additional factors warrant further investigation.

**FIGURE 18:**
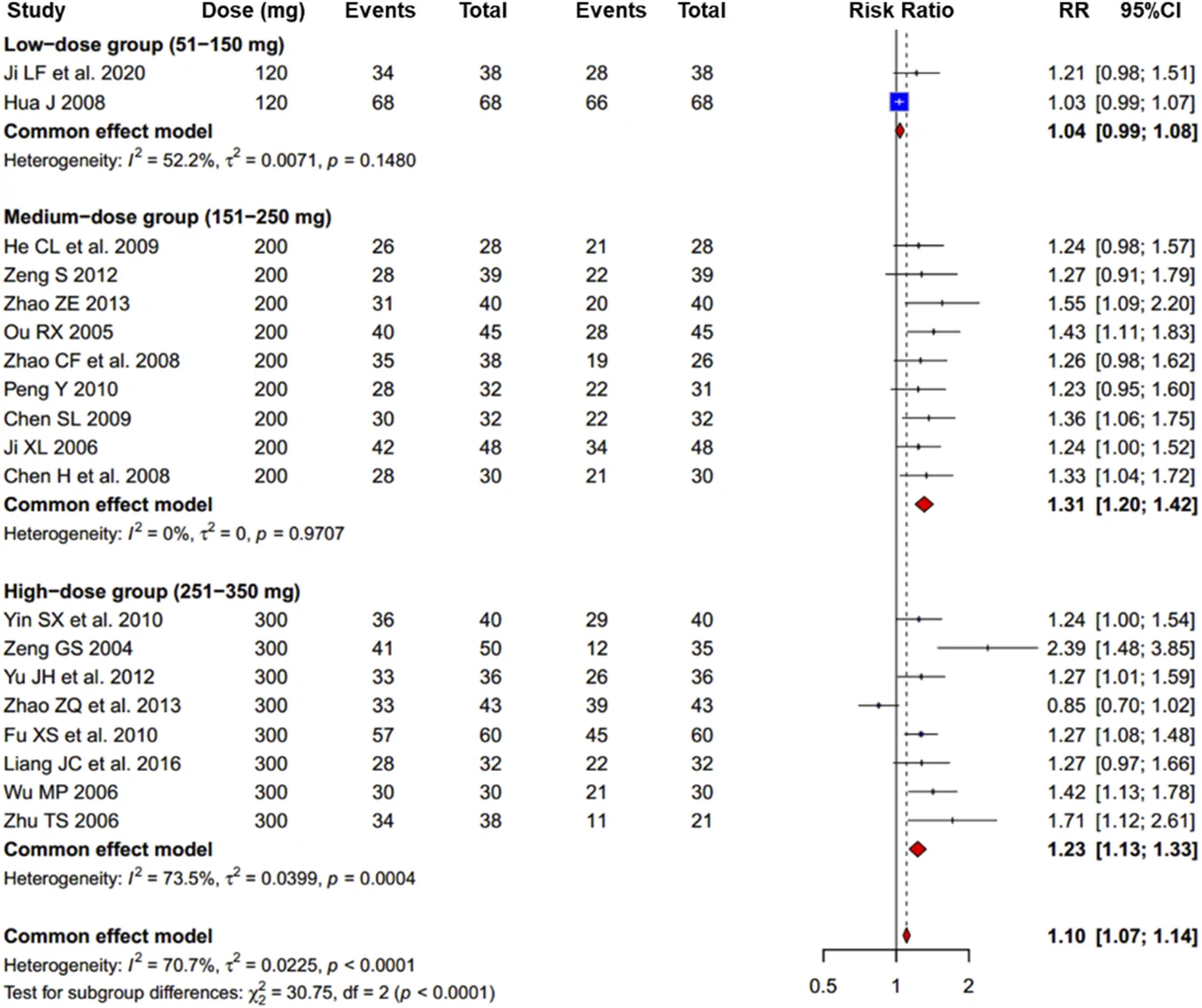
Forest plot for subgroup analysis comparing differences in overall response rates across different doses of berberine.

#### 3.3.11 Quality of evidence

According to the GRADE approach, the quality of evidence from RCTs may be downgraded for five reasons: risk of bias, imprecision, inconsistency, indirectness, and publication bias. The GRADE assessment of evidence quality (Supplementary Table S2) indicates that although all included studies were initially rated as high-quality RCTs, the overall evidence level was downgraded to low. This downgrade was attributed to incomplete methodological reporting in most studies, potential publication bias, and imprecise effect estimates. Therefore, conclusions regarding intervention effectiveness should be interpreted with caution, and further high-quality research is needed to confirm these findings.

## 4 Discussion

This work comprehensively evaluated the efficacy and safety of berberine combined with brain-gut axis-targeted drugs for treating IBS-D through systematic review and meta-analysis. Results demonstrated that this combined treatment significantly outperformed controls by improving the overall response rate (OR = 4.19), alleviating symptoms including diarrhea, abdominal pain, and bloating, and reducing recurrence rates (OR = 0.28), with no serious adverse events reported. However, subgroup analyses revealed heterogeneity in efficacy according to dosage, treatment duration, and combination regimens. Furthermore, the included studies exhibited a high risk of bias, leading to a low-GRADE evidence quality rating. This work elucidated potential mechanisms underlying the synergistic effects of combination therapies based on IBS-D pathophysiology, analyzed the clinical implications of heterogeneity, and critically assessed the strengths and limitations of existing evidence to guide clinical practice and future research.

The therapeutic advantage identified in this meta-analysis fundamentally arises from the precise targeting of the complex, multilayered pathophysiological network underlying IBS-D by the combination therapy. This approach enables bidirectional, closed-loop regulation of the central brain-gut axis. The pathogenesis of IBS-D involves a vicious cycle between peripheral intestinal dysfunction and abnormal central nervous system processing (Mayer et al., 2014; Ford et al., 2017).

Targeted brain-gut axis medications, including 5-HT_3_ receptor antagonists and tricyclic antidepressants, primarily act through a top-down regulatory pathway. By modulating key neurotransmitters such as serotonin and noradrenaline, these agents act on central and peripheral receptors to reduce visceral hypersensitivity and normalize abnormal gastrointestinal motility (Ford et al., 2017). However, these drugs have limited effects on the intestinal microenvironment, which may explain their ineffectiveness in some patients (Rivkin and Rybalov, 2016).

Berberine pharmacologically compensates for this limitation by primarily exerting therapeutic effects via a bottom-up mechanism. Its broad-spectrum antibacterial activity and modulation of microbial communities contribute to restoring gut microbiota homeostasis (Kim et al., 2020). Berberine exerts anti-inflammatory effects by inhibiting key signaling pathways, including NF-κB, thereby alleviating low-grade intestinal inflammation (Kim et al., 2020; Takahara et al., 2019; Gong et al., 2020). It promotes repair of damaged intestinal mucosal barriers and reduces enteric antigen translocation by upregulating tight junction protein expression. Its antioxidant properties further enhance tissue repair by mitigating inflammation and oxidative stress (Wang and Zhang, 2018). Additionally, berberine reduces intestinal secretion by inhibiting the cystic fibrosis transmembrane conductance regulator (CFTR) chloride channel and modulating the adenylate cyclase pathway. Concurrently, it regulates gastrointestinal motility (Chen et al., 2014; Menees et al., 2012). The effects berberine extend beyond the gut, influencing the central nervous system via the microbiota-gut-brain axis. Its analgesic properties may involve nitric oxide (NO) pathways, antioxidant effects, and anti-inflammatory actions (Kim, 2015; Kim and Kim, 2013). Furthermore, berberine treatment has been linked to reductions in IBS symptom severity and in anxiety and depression scores (Chen et al., 2015), suggesting a regulatory role in the neuroendocrine system.

Therefore, the superiority of combined therapy arises not from a simple additive effect but from the establishment of a closed-loop regulatory mechanism characterized by peripheral source reduction and central desensitization. “Source reduction” refers to action of berberine at the intestinal level in diminishing abnormal microbial flora, inflammatory stimuli, and barrier damage, thereby reducing the intensity of abnormal signals transmitted to the central nervous system at their source. “Desensitization” refers to the process by which brain-gut axis medications centrally reduce visceral sensory thresholds, modulate emotional responses, and enhance descending inhibitory control. The synergy of these approaches forms an integrated model combining top-down and bottom-up strategies, enabling a more comprehensive and multidimensional intervention of the brain-gut axis. For example, Wu (2006) study demonstrated significant therapeutic effects by combining oral berberine with Shenling Baizhu Powder, a formulation aimed at invigorating qi, strengthening the spleen, dispelling dampness, and arresting diarrhea to regulate the brain-gut axis. This underscores the unique advantages of multi-component, multi-pathway synergistic mechanisms.

Although primary efficacy endpoints demonstrated high consistency, in-depth subgroup analyses identified key sources of heterogeneity, providing critical evidence to optimize clinical protocols.

Firstly, therapeutic efficacy exhibited a clear dose-dependent relationship. Analysis showed that combination therapy produced consistent and significant efficacy only when berberine doses exceeded 150 mg per administration. The medium- and high-dose groups demonstrated significant efficacy, with RRs values ranging from 1.31 to 1.32. In contrast, the low-dose group (≤150 mg) did not demonstrate significant efficacy, showing an RR of 1.08. This suggests the existence of an effective dose threshold for berberine in modulating the complex pathological network of IBS-D. Doses below this threshold may lack sufficient pharmacological potency to exert significant therapeutic effects. These findings provide crucial dosimetric evidence for formulating effective clinical prescriptions, supporting the recommendation to use adequate berberine doses in combination regimens.

In addition, treatment duration is a significant factor influencing outcome stability. Subgroup analysis revealed extremely high heterogeneity (*I* ^2^ = 93.1%) in the short-term (2-week) treatment subgroup, whereas subgroups with treatment durations of 3 three weeks or more exhibited substantially lower heterogeneity. These findings indicate that short-term interventions may be insufficient to fully realize the therapeutic effects of combination therapy, particularly peripheral source-reduction mechanisms mediated through pathways such as the microbiota-immune axis, resulting in inconsistent efficacy assessments. Therefore, a minimum treatment duration of four weeks is recommended for clinical intervention and efficacy assessment to ensure stable and reliable therapeutic outcomes.

Moreover, the composition of combination therapy regimens is a primary source of heterogeneity. Heterogeneity was predominantly concentrated in the “berberine + probiotics” subgroup (*I* ^2^ = 93.6%), whereas subgroups combining antidepressants, gastrointestinal regulators, or Jianpi compound decoctions showed good internal consistency. This heterogeneity likely stems from substantial variations in probiotic strains, viable bacterial counts, and formulations used across different research institutes, making their biological effects difficult to compare. Given the pronounced heterogeneity in probiotic preparations among studies examining “berberine plus probiotics,” particular caution is warranted when interpreting their conclusions. Future studies should rigorously standardize probiotic formulations and provide comprehensive reporting of their composition and dosage. In contrast, combining berberine with brain-gut axis drugs that have well-defined mechanisms of action tends to yield more predictable and reproducible therapeutic outcomes.

When interpreting the positive conclusions of this work, it is essential to consider the limitations of the underlying evidence base. According to the GRADE framework, the quality of evidence for the primary outcome was rated as low. The primary reasons for downgrading were that most included studies exhibited a high risk of bias due to inadequate blinding, particularly regarding participant and investigator blinding. This limitation may introduce substantial performance bias in IBS-D research, where patient-reported subjective symptoms constitute primary outcomes, potentially leading to overestimation of treatment effects. Moreover, although statistical tests for publication bias yielded inconsistent results, trim-and-fill analysis suggested the possible presence of unpublished small-sample studies with negative findings. Although this did not change the overall direction of the conclusions, it may have led to a more conservative estimate of the effect size.

Therefore, when translating the findings of this study into clinical decision-making, a cautiously optimistic approach is warranted. Current evidence supports that for patients with IBS-D who respond poorly to conventional monotherapy, particularly those presenting with significant anxiety or depressive symptoms, or exhibiting gut microbiota dysbiosis and signs of low-grade inflammation, employing a targeted brain-gut axis therapeutic strategy at full dosage (berberine >150 mg per dose) and full course (≥4 weeks) represents a reasonable and promising option. In clinical practice, combination regimens with higher evidence consistency (such as those incorporating specific brain-gut axis medications) should be prioritized. Patients should be fully informed of the limitations of the current evidence, with therapeutic efficacy and adverse events closely monitored throughout the treatment course.

## 5 Conclusion

This meta-analysis evaluated the efficacy and safety of berberine combined with brain-gut axis-targeted drugs for the treatment of IBS-D. The results indicate that combination therapy significantly improves overall efficacy, alleviates diarrhea symptoms, and reduces the risk of recurrence, with good tolerability. The synergistic effects of berberine may be attributed to its multi-target modulation of the gut-brain axis, involving coordinated regulation of both peripheral intestinal pathways and central nervous system functions. For patients with inadequate response to monotherapy, using berberine in combination with brain-gut axis modulators at an adequate dose and duration may be considered. Although the current evidence is limited by the methodological quality of the original studies, the consistency of effect estimates and underlying pharmacological rationale provide supportive evidence for these findings. High-quality, well-designed clinical trials are still needed to further validate these findings and clarify their optimal clinical applications.

## Data Availability

The datasets presented in this study can be found in online repositories. The names of the repository/repositories and accession number(s) can be found in the article/Supplementary Material.
