# Supplementary material for: A meta-analysis of berberine combined with brain-gut axis-targeted agents for diarrhoea-predominant irritable bowel syndrome
Source: Front Pharmacol. 2026 Sep 30;17:1795170. doi: 10.3389/fphar.2026.1795170 (PMC13662641; doi:10.3389/fphar.2026.1795170)
Supplement: Supplementary file 1 [file Image1.pdf]

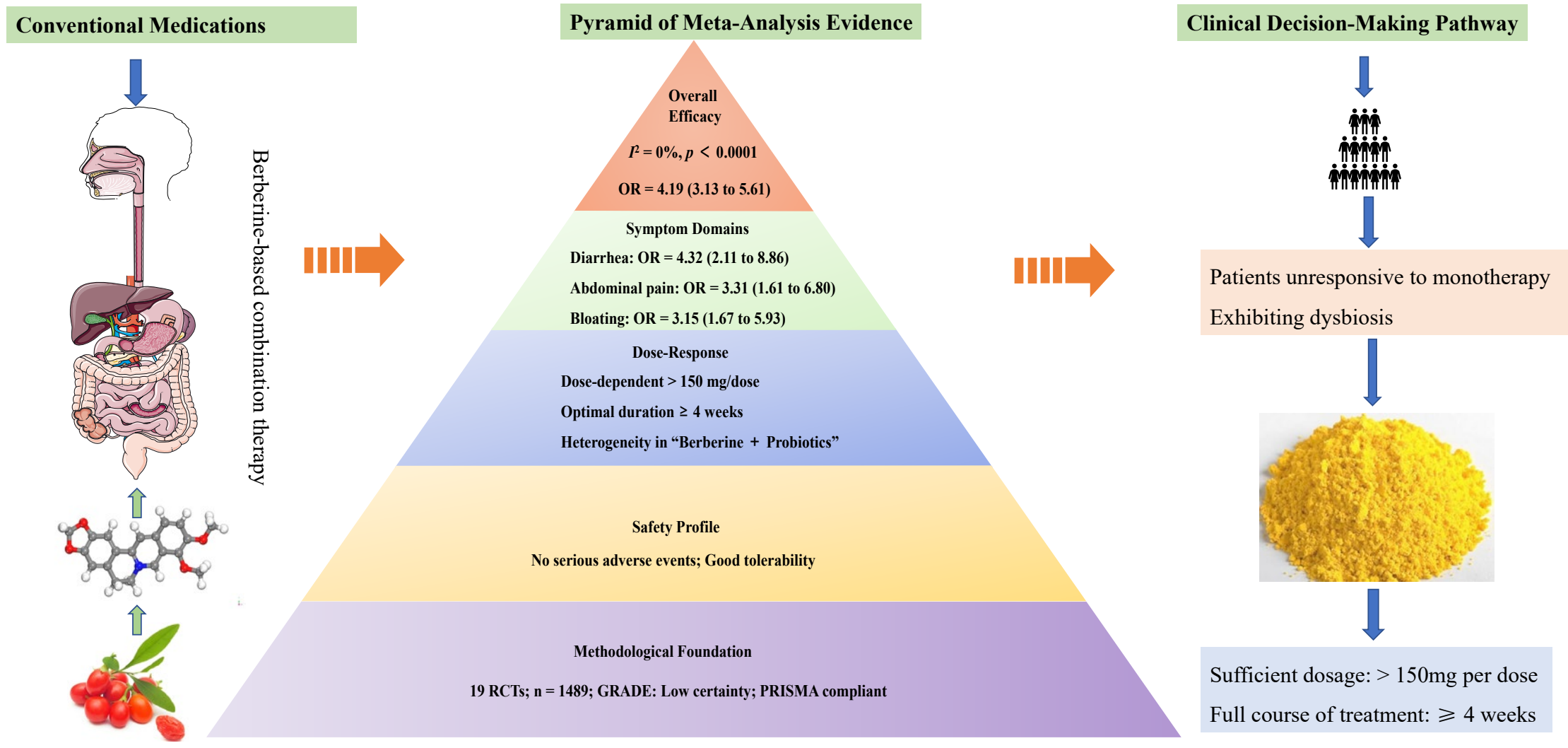
Conventional Medications
Berberine-based combination therapy
Pyramid of Meta-Analysis Evidence
Overall Efficacy
I^2 = 0%, p < 0.0001
OR = 4.19 (3.13 to 5.61)
Symptom Domains
Diarrhea: OR = 4.32 (2.11 to 8.86)
Abdominal pain: OR = 3.31 (1.61 to 6.80)
Bloating: OR = 3.15 (1.67 to 5.93)
Dose-Response
Dose-dependent > 150 mg/dose
Optimal duration ≥ 4 weeks
Heterogeneity in "Berberine + Probiotics"
Safety Profile
No serious adverse events; Good tolerability
Methodological Foundation
19 RCTs; n = 1489; GRADE: Low certainty; PRISMA compliant
Clinical Decision-Making Pathway
Patients unresponsive to monotherapy
Exhibiting dysbiosis
Sufficient dosage: > 150mg per dose
Full course of treatment: ⩾ 4 weeks
